# Building sustainable and scalable peer-based programming: promising approaches from TESFA in Ethiopia

**DOI:** 10.1186/s12978-021-01304-7

**Published:** 2022-06-13

**Authors:** Pari Chowdhary, Feven Tassaw Mekuria, Dagmawit Tewahido, Hanna Gulema, Ryan Derni, Jeffrey Edmeades

**Affiliations:** 1 Health Equity and Rights Team, CARE USA, Atlanta, USA; 2 Research and Community Service, Addis Continental Institute of Public Health, Addis Ababa, Ethiopia; 3 Global Public Health and Health Policy, Addis Continental Institute of Public Health, Addis Ababa, Ethiopia; 4 Demografix, Arlington, USA

**Keywords:** Adolescent sexual and reproductive health, Peer education, Social norms, Child marriage, Sustainability, Scale

## Abstract

**Background:**

Girls in Ethiopia’s Amhara region experience high rates of child marriage and are less able to negotiate sex or use family planning. Seeking to improve their lives, CARE’s TESFA programme delivered reproductive health and financial savings curricula to married girls via reflective dialogues in peer-based solidarity groups. From 2010 to 2013, 5,000 adolescent girls participated via three intervention arms: sexual and reproductive health, economic empowerment, and a combination of both. At end-line, participants reported improvements across health and empowerment outcomes. Four years post-TESFA, 88% of groups reported meeting without continued assistance from CARE. Some original participants had created new groups based on the TESFA model, and some girls not recruited for TESFA spontaneously replicated it to create their own groups. However, questions remained about what had contributed to this organic sustainment and scale-up of groups.

**Methods:**

This 2018 study investigated factors affecting sustainability and scale-up of peer solidarity groups through a systematic mapping of TESFA groups across five *woredas* (districts) and interviews with key stakeholders. Data were collected from 39 focus groups with active and dissolved Girl Groups, Social Analysis and Action groups, and girls’ husbands and from 29 in-depth interviews with group facilitators and community health workers across three districts. Data were coded and analyzed per grounded theory principles.

**Results:**

Changes in reproductive health knowledge and specific behaviours, such as contraceptive use and institutional delivery, were maintained 5 years after the intervention ended. Group connectedness, spousal support, integration of holistic community platforms, and opportunities for financial independence were found to be important for group sustainability. Observed changes in TESFA girls’ confidence to negotiate and assert their rights, hopes of improved mobility, and the promise of economic opportunity commonly inspired spontaneous replication of groups. Recommendations for future peer-based programmes include creating environments of solidarity and holistically engaging intervention communities.

**Conclusion:**

By increasing knowledge of and access to reproductive health services, TESFA mitigates some of the harmful effects of child marriage. The maintenance and organic replication of groups suggest that TESFA provides a successful, scalable and sustainable tested model for reproductive health program delivery through peer-based solidarity groups.

**Supplementary Information:**

The online version contains supplementary material available at 10.1186/s12978-021-01304-7.

## Plain Language Summary

In Ethiopia, married girls are a particularly vulnerable adolescent population because of the many health risks associated with early marriage. Peer-based approaches are a common strategy to improve sexual and reproductive health knowledge and behaviours for marginalized groups. Critiques of these approaches are that they lack sustainability without ongoing investment and have no lasting impact on health behaviours. CARE’s TESFA programme, a peer-based approach with married adolescent girls in South Gondar, was found to be effective in creating long-term behavior change related to uptake of family planning and rates of institutional delivery. Some 88% of groups who received the TESFA curriculum were still meeting 4 years after the programme had concluded. Some participants even created groups of their own, scaling TESFA beyond the original audience, suggesting a potential approach to wider scale-up. To explore lessons learned around scale and sustainability, this study interviewed TESFA participants and stakeholders.

Improvements in behaviours related to contraceptive use, institutional delivery, and girls’ agency in reproductive decision-making were sustained over time regardless of group status. Factors that affected the sustainability and scale-up of groups included solidarity among group members, support from husbands and community members, ongoing savings and loan structures, flexibility of group processes, and availability of relevant curriculum topics. Through an analysis of these findings, this paper discusses recommendations for sustainable and scalable peer-based programme design via existing Ethiopian community structures. Using TESFA principles as a model, practitioners could design and implement scalable and sustainable peer-based programming for adolescents sexual and reproductive health.

**Supplementary Information:**

The online version contains supplementary material available at 10.1186/s12978-021-01304-7.

## Background

Despite gains made towards reducing child and early marriage in Ethiopia, recent data shows that over 40 percent of women aged 20–24 years are married before the legal age of 18 [1]. In the Amhara region of Ethiopia, the median age of a girl at marriage is 15.7 years [1]. Early marriage can increase physical, physiological, and emotional risks of adolescents, especially for girls [2–4]. Once married, adolescent girls often experience solitary and routine lives with their husbands and mothers-in-law maintaining much of the decision-making power. Especially in rural areas, child brides typically drop out of school, are isolated to domestic tasks, and have increased risk of intimate partner violence [5]. Married adolescent girls often experience health complications due to early pregnancy and childbearing, limited reproductive health knowledge and access to health services, and a low ability to negotiate sex or use of family planning [6, 7]. Together these conditions and community norms create an environment of intense vulnerability for married adolescent girls.

To meet the unique needs of married adolescent girls in Ethiopia, CARE created TESFA (Towards Economic and Sexual/Reproductive Health Outcomes for Adolescent Girls). TESFA, meaning ‘hope’ in Amharic, was one of a few global efforts focused solely on married girls and how best to support their transition to adulthood. It sought to mitigate the harmful effects of child marriage by creating opportunities for girls to acquire reproductive health knowledge and engage in economic activity through peer-based solidarity groups. *Equb*, informal savings and credit schemes for indigenous communities, have long existed in Ethiopia but traditionally exclude married adolescent girls [8]. Building on CARE’s Village Savings and Loan Association (VSLA) model, TESFA organized girls into “Girls Groups” (GG) who received customized sexual and reproductive health (SRH) curriculum, economic empowerment (EE) curriculum, and life skills and communication training. Relative to *equb*, TESFA’s VSLA is more structured with consensus-based rules and implementation regulations for loan and social fund components. It uniquely catered to girls' needs by promoting solidarity and enabling groups to become self-supporting. Girls Groups were led by trained and supported peer facilitators, not educators but learners themselves, who used participatory tools to encourage dialogue, reflection, critical thinking, and active learning among group members. TESFA groups afforded girls a safe place to meet and build a supportive network with other married adolescents. Recognizing that girls’ lives cannot be affected without changing societal norms, TESFA also engaged village elders, religious leaders, community health workers, mothers-in-law, and girls’ husbands into “Social Analysis and Action (SAA) groups”. Through reflective dialogues in support of adolescent girls’ access to information and services, SAA groups sought to address the social barriers faced by married girls and create enabling environments for positive health and economic outcomes through a gender-transformative approach and process [9].

Launched in 2010, TESFA was initially implemented in two *woredas* (districts) in the South Gondar zone of the Amhara region [10]. (Additional file 2: Figure S1 depicts the timeline of CARE's TESFA program). From 2010 to 2013, 5,000 married girls aged 10–19 years participated in TESFA, divided into three intervention arms: one focused on SRH, one on EE, and one combined SRH and EE curriculum. Although peer-facilitated programmes have been deemed effective in empowering otherwise hard-to-reach populations to increase their knowledge, there is limited evidence of their impact on behavior change. Four systematic reviews of peer education programmes across different countries concluded that the interventions had an equivocal impact on adolescent SRH behaviours [11–14]. TESFA’s end-line evaluation in 2013 found economic, health and social improvements in participants’ lives [15]. Participants reported gains in communication between girls and their husbands, decreased levels of gender-based violence, improved mental health among participating girls, increased investment in productive economic assets, and increased social capital. The evaluation found increases in participants’ knowledge and uptake of family planning, knowledge of antenatal care, knowledge of sexually transmitted diseases, understanding of fertility, household decision-making, and general mobility [15]. Michielsen et al. posit that integrating peer education into holistic interventions and employing peer educators to sensitize and refer participants to experts/services could result in increased effectiveness [16]. TESFA’s design accounted for both these components—married adolescent girls participated in peer-facilitated dialogues with visits from and referrals to health workers, while their husbands, mothers-in-law, and community members simultaneously participated in discussion groups intended to influence norms at the community level. Per the evaluation findings, TESFA was effective in mitigating some of the harmful effects of child marriage by increasing knowledge and use of reproductive health services.

Still, TESFA’s sustainability and scaling potential were unknown. To explore this, the Addis Continental Institute of Public Health (ACIPH) conducted an ex-post evaluation in Farta district in 2017, 4 years after TESFA’s implementation period had ended [17]. Some 88% of surveyed groups were found to still be active and using the SRH and EE curricula. There was also auto-replication i.e. individuals who had not originally participated had joined TESFA groups and new groups inspired by and based on the original TESFA model had been developed [17]. For the latter, findings revealed two types of new groups—those started by original participants, and those started by girls not originally recruited for TESFA who had spontaneously replicated the model to create their own groups [17]. These findings were unexpected. Peer-based approaches like TESFA are a universal strategy for improving knowledge and behaviours in marginalized populations, and a common critique is that they lack sustainability without ongoing investment [11, 18]. Not only had Girls Groups self-sustained 4 years post program close-out but they had spread through replication, disseminating the programme approaches and outcomes beyond TESFA’s original intervention areas. To understand what contributed to this organic sustainment and scale-up of TESFA, CARE and ACIPH conducted an investigative research study in 2018. This paper shares those results and outlines recommendations for sustainable and scalable adolescent sexual and reproductive health peer-based programme design.

## Methods

### Study design

This investigative research study was designed to assess the facilitators and barriers of sustainability of peer-facilitated groups, and the implications these have for scale-up. This study included a systematic mapping of the status of TESFA groups across five *kebeles* (villages), and a qualitative investigation with programme participants of the conditions required for success, sustainability and scale-up of Girls Groups. Based on earlier evaluation findings suggesting participants who underwent the combined SRH and EE curriculum experienced the greatest overall benefits, this study only included villages that implemented the combined programming arm.

### Setting, sampling and recruitment

This study was conducted in May 2018 in five villages in the Lay Gayint district within the South Gondar zone in the Amhara region. The villages (Mekuwabia, Zuramba, Addis Amba, Yedero and Guna) were selected because they were part of the combined programming arm, within the TESFA implementation area, and at a close-enough distance to facilitate travel back and forth. Mapping of groups was carried out in all five villages, pre-testing of data tools was done in Yedero, and data collection was conducted in Mekuwabia, Zuramba and Addis Amba.

To determine the status of each original TESFA and new group, CARE staff met with community gatekeepers and group facilitators in each village. Groups were categorized as dissolved (an original TESFA group that had not met for six months prior to the study), active (an original TESFA group still meeting on a regular basis), or auto-replicated (a group of married girls that used a TESFA-like savings model or identified TESFA as inspiration for their group’s creation). Mapping results were used to ensure representation across all participant groups and informed the choice of the three villages. CARE staff then traveled to the relevant villages to inform the community about the call for participants and employed purposive sampling techniques to recruit girls, girls’ husbands, girls’ mothers-in-law, religious leaders, and health workers for participation.

### Data collection

Data were collected by research assistants (RAs) through semi-structured focus group discussions and in-depth interviews, field notes and daily summaries. CARE and ACIPH staff developed focus group discussion and in-depth interview tools in English, and then translated them to Amharic. During the RA training and field pre-testing, all the tools were refined for colloquial and everyday Amharic language (See Additional file 1 for some of the data collection tools).

This study gathered data from 320 participants through 39 focus group discussions and 29 in-depth interviews. For participant characteristics, see Table 1.

**Table 1:** Number and characteristics of focus group discussion (FGD) and in-depth interview (IDI) participants

Target Group	Group Status	Number of FGDs	Number of IDIs	Gender Distribution	
				Female	Male
TESFA Girls Group	Dissolved	3		12	
	Active	9		36	
Non-TESFA Girls Group	Auto-replicated	3		27	
TESFA SAA Group	Dissolved	3		16	14
	Active	6		39	26
Non-TESFA SAA Group	Auto-replicated	3		24	7
Girls Group facilitators			12	12	
SAA facilitators			11	5	6
Husbands of TESFA Girls Group members		6			38
Health workers			6	5	1
Development agents		6		14	33
Total		39	29	195	125

Each focus group had a facilitator and a note taker. Each interview had a sole facilitator. Girls Groups and husband groups were led by a female and male facilitator, respectively. Interviews and focus groups were held in enclosed/private spaces within villages. All sessions were audio recorded. RAs translated the content into English during verbatim transcription. At the end of each field day, the full research team convened to debrief and ensure consistency of data collection methodology.

### Data analysis

Using grounded theory principles, transcripts were repeatedly and carefully reviewed to inductively develop a coding structure. The first round of review explored participants' general perceptions of the TESFA model and potential areas for improvement. Based on that reading and the study’s objectives, an initial set of codes was developed and then honed through further data review. The next round of transcript review focused on descriptions of individual characteristics or conditions related to maintaining, dissolving, or replicating a group. Subsequent line-by-line coding focused on identifying facilitators and barriers for each of those group types, and potential pathways to scale. All coding was done in OpenCode 4.2, a software tool designed for coding qualitative data. Following an iterative coding and memo process, two of the authors jointly developed a codebook which was then validated by a third author. Once the full data set was coded, data were further sorted by broader themes that had emerged.

### Ethical considerations

ACIPH’s Institutional Review Board approved this investigative research study (ACIPH/IRB/006/2018). Care was taken during study design to ensure participant confidentiality, safety, and comfort. Support letters were sought from relevant government offices. Study participation was entirely voluntary, and participants were free to opt out of specific questions or withdraw from the study at any time. All participants engaged in informed verbal consent. No personal identifiers such as names and titles were recorded.

## Results

Study results are organized into the following broad sections: health and social impacts, and factors impacting sustainability and scale.

### Health and social impacts

Participants reported continued improvements in SRH knowledge, uptake of family planning, and healthy pregnancy practices. Across all villages, health workers reported sustained increases in three behavioural areas since TESFA’s inception: contraceptive use, girls’ agency in reproductive decision-making, and rates of institutional delivery.

“*Before, parents wouldn’t take a girl to the health center due to habit of prolonged labor at home. Now they do. Now we also limit to 2-3 children.*”—Health worker

For some SRH behaviours, health workers reported high initial adoption post-TESFA but slowed maintenance of positive behaviour change over time. These included child-spacing, and antenatal and postnatal care visits. Girls Group participants shared that TESFA’s SRH curriculum and life skills training were critical in increasing their awareness of SRH practices and their confidence to assert their reproductive rights. Participants reported improved couples’ communication on SRH matters such as sexual consent and use of contraception. At a community level, most participants discussed increased support and acceptance of family planning and health service access. Contraceptive decision-making was repeatedly cited as a long-term sustained shift in the community.

“*Earlier if their husbands said no, the girls would not use contraception. After a long process, there is less male influence. The girls decide themselves.*”—Health worker

Most husbands were initially uncomfortable with their wives’ involvement in TESFA and described how prior to TESFA, it was normal and expected that a husband alone decides when to have sex and how many children to have. The majority said their opinions began to change following their participation in SRH sessions within their SAA groups, and continued to because of ongoing community dialogues.

“*I didn’t want to lose my power. A man is supposed to be the head of the family. But I’ve seen that I should know more about my health and talk to my wife about how many children I want.*”—Husband, SAA participant

Other SAA participants noted that, although sometimes challenging, having mixed-gender groups facilitated the sharing of different perspectives and encouraged them to shift SRH behaviours and norms to find common ground. An area where this appears to have unequivocally been achieved across TESFA villages is decreased community acceptance of child and early marriage. GG participants repeatedly discussed their intentions to “save” their younger sisters and future daughters from early marriage.

“*We are teaching our younger sisters not to face our fate. I will let my daughter choose when to get married. I prefer she gets married after the age of 20 but this will be only her decision, not mine or anybody else’s.*”—GG participant, age 22

Participants from every target group shared stories of instances where community members had advocated for or acted towards delaying marriage of young girls. There was widespread hope among participants that by practicing and spreading the TESFA lessons to younger girls and community members, these outcomes would be sustained. Many SAA participants described continuing to take actions to reduce incidences of early marriage out of a sense of responsibility towards TESFA and the community. When asked whether TESFA’s curriculum had specifically contributed to this, GG and SAA participants said learning about the short-term risks and long-term physiological impacts of adolescent pregnancy influenced their views on early marriage and childbearing.

### Factors impacting sustainability and scale

*Connection and mobility:* Connectedness with similar-aged girls was the most influential element for group maintenance and the greatest impetus for group replication. Participants repeatedly cited the importance of feelings of solidarity and sisterhood to their continued engagement with their groups and TESFA in general.

“*I appreciate our closeness. Our group is special because it has love. Because we love each other, we miss each other until we meet again*.”—GG participant, age 17

Freedom of movement granted by participation in a Girls Group was identified as impactful to group sustainability. Participants discussed how the TESFA model afforded them mobility and greater independence and shifted community norms on girls’ freedom to leave their homes unattended.

“*There is a saying: women go to the kitchen and men go out. We were hidden. After TESFA, I feel freer. I can go out. I can speak up. I can live*.”—GG participant, age 19

When asked how these feelings of connectedness and freedom relate to group sustainability, participants described hopes of continued programming that not only ensured their ability to connect and move freely but also institutionalized these norms for future generations of girls in their communities. SAA participants also described experiencing renewed connections with each other and the community in the form of feelings of responsibility and ownership over supporting the girls and creating social change.

*Economic opportunity:* Every participant named TESFA’s EE curriculum as a crucial component for long-term group sustainability. For auto-replicated groups, observing the impacts of TESFA girls’ saving practices was the second most cited reason to scale TESFA into their own communities. Participants labelled TESFA’s structure and girl-specific savings and income generation practices as revolutionary to their lives.

“*It changed everything. There was a power imbalance. I had nothing so I had no say with my husband. Now I own resources and can plan for emergencies. I sell cattle and know the prices. There is open discussion about money*”—GG participant, age 18

Because SRH and EE curriculum delivery had ended 5 years earlier, original groups continued to meet primarily on the grounds of savings. Girls Groups that remained active often mentioned motivations related to economic independence and were typically found to have followed the VSLA approach more robustly than groups that dissolved. In Zuramba, some participants reported using TESFA’s economic lessons to join the Amhara Credit and Savings Institute to access credits and maintain a formal savings process. Those that didn’t join *equb* structures post-TESFA said that without CARE’s presence, support from SAA groups was important to sustaining a savings culture and a social environment that normalized group continuity.

“*We told husbands it was good for [GG] to keep meeting. They could make money for their families; that’s better for their children*”—Village elder, SAA participant

This observed importance of economic opportunity to group sustainability and scale appeared to also extend to dissenters. Husbands who opposed their wives’ continued participation in TESFA groups post-CARE’s implementation often considered groups pointless without a microfinance component from which their families could benefit.

*Household relationships:* Girls and their husbands commonly discussed improvements in their relationships, communication, and joint decision-making following participation in TESFA. Participants reported increased support for girls’ engagement in the community, increased equity in home-making, and a sharing of responsibilities. Some of the husbands shared that while they were initially hesitant to accept it, they now appreciate what TESFA has achieved.

“*I thought I would lose something if my wife was earning and talking to others. But we are a better couple now. She is happy and we have more money. Maybe there is more we can do together.*”—Husband, SAA participant

Girls reported their confidence to take active roles in household matters and negotiate responsibility was bolstered by their continued participation in groups because they received encouragement from group members. Auto-replicated GG participants cited a desire to have happier household environments and be better self-advocates with their family as a reason for creating their groups. Relationship improvements were also widely reported between girls and their mothers-in-law by participants of original and auto-replicated groups.

“*My husband’s mother was very controlling. After TESFA, I have an easier relationship with my daughter in law*”—Mother-in-law, SAA participant

Another way in which participants connected their improved domestic situations to their interest in sustaining and scaling groups was a belief that continued participation in programming would affect further positive change.

*Curriculum content:* Because TESFA’s original SRH and EE curricula were designed for a defined implementation period of 4 years, they didn’t include additional content to account for continuation of groups beyond that time. Participants reported experiencing boredom with and fading interest in repeated discussions of the same curriculum topics after several years.

“*We didn’t know what else to talk about. We had already gone through [the curriculum] many times. It was always the same.*”—GG participant, age 21

Participants repeatedly identified a need for refreshed or new curriculum on non-savings related topics to ensure continuation and long-term sustainability of programme outcomes. A specific example of a topic that participants cited was business and entrepreneurship training. Some hypothesized that extension of the curriculum would supplement their savings structure to support group maintenance and encourage continued community support. Groups that sustained eventually ran out of discussion topics and gradually shifted their primary focus towards the savings component. Despite wanting to continue meeting, the lack of new curriculum content resulted in dissolved groups feeling like they didn’t have much reason to once they had shared out all their savings. Without the TESFA curriculum to work with, auto-replicated groups sometimes developed their own TESFA-like conversation topics but reported finding it even tougher to sustain beyond that without the savings aspect. Some participants noted that they were looking to their group facilitators for leadership, but facilitators were not always well-suited to sustain groups.

“*First I was managing by revisiting old topics. I asked the health worker to talk to our group, but after that, I didn’t have other ideas.*”—GG facilitator, age 23

SAA participants also acutely felt the effects of a lack of new discussion content, and instead oriented themselves around action plans to support girls in their communities. This included spreading awareness of the detriments of early marriage and benefits of family planning.

*Support from husbands:* Some dissolved GG participants reported that their greatest barrier to sustaining their groups was a lack of continued support from their husbands and families.

“*Our facilitator left because her husband was offensive to the group saying continuing was a waste of her time, even after four years*”—GG participant, age 19

Without an institutional presence or the promise of economic gain, some husbands did not believe that other benefits of group involvement warranted continued participation from their wives. Even if they had experienced improvements in their household relationships, participants discussed how their freedom of choice and movement changed once CARE’s follow-up had ended. Participants also discussed how the conclusion of SAA groups, which included husbands, at the end of TESFA, made it difficult for their husbands and family to understand why the Girls Groups would continue to meet.

“*[TESFA] was over. There’s no need to meet anymore.*”—Husband, SAA participant

Some dissolved GG and SAA participants suggested that their husbands’ negative perceptions of the continuation of groups may stem from concerns around not knowing what girls would be discussing with their peers. Although there was initial acceptance of girl-only groups, this changed once Girls Groups were unaccompanied by simultaneous SAA groups. To address this withdrawal of husbands’ support, participants suggested the creation of a long-term accountability mechanism by implementers or the local government. Participants shared that a continued external presence, even at a low frequency or remotely, positively influences the community’s social investment in their girls, and would alleviate fears that norms would return to the status quo after programmes end.

*Adaptations to groups:* The TESFA model afforded groups some flexibility in making adaptations to the programme design. While some adaptations were perceived as helpful, such as monthly meeting frequency and flexible loan repayment schedules, participants reported certain adaptations that their group had adopted as significant barriers to maintaining their commitment to their group. These included financial penalties for various “offences”, such as lateness and missed meetings.

“*My group ended because for different wrong doings they would impose penalties that felt like an extra burden.*”—GG participant, age 20

Participants shared that groups with stronger dynamics i.e., connectedness and friendship, were generally more likely to adapt in ways that were beneficial and rewarding to all the group members.

## Discussion

This study highlights the components necessary for the success, sustainability, and scalability of a peer-based approach for adolescent sexual and reproductive health. In investigating the effectiveness, sustainment, and spontaneous replication of TESFA’s peer-based solidarity groups 5 years post programme close-out, the study team identified three encouraging findings. First, positive changes in behaviours promoted and supported by the programme had been maintained. Second, original groups remained active through the peer-based reflective dialogue and savings model established during the programme. Third, individuals spread and created new groups modeled on the programme in response to demand from peers in nearby communities. Each of these had occurred without outside investment.

Across participants of the original TESFA Girls Groups, this study found sustained improvements in girls’ inclusion and agency in reproductive decision-making, use of contraception, and institutional delivery. Changes in other measured SRH behaviours, such as child spacing, faded with time. These persisted changes in behaviour have implications for existing critiques in the literature of the effectiveness and sustainability of peer-based approaches to adolescent sexual and reproductive health (ASRH). Five systematic reviews of peer-led sexual health education between 2008 and 2020 have found varying evidence of effectiveness in promoting behaviour change [11–14, 19]. Despite seeing improvements in SRH knowledge in their meta-analysis of 13 programmes, Kim et al. surmised that peer-led education does not result in improved SRH outcomes among adolescents [11]. In their review of 15 peer-led interventions, Sun et al. concluded that the evidence for effectiveness in behaviour change is lacking [12]. However, a review and meta-analysis of 60 peer-led HIV prevention education programs by He et al found evidence for effectiveness and determined peer education to be useful for long-term impact on behaviour change [19]. TESFA’s effectiveness for long-term behaviour change is supported by the results of this investigative research. It is worth noting that most of the literature on effectiveness of peer programmes are focused on peer-led education, but TESFA employed a model of peer-facilitated reflective dialogues intended to promote solidarity between group members. Study results showing the strong influence of group closeness and connectedness to sustainment and scale-up of TESFA groups suggest that the social environments within peer groups are important considerations for long-term change. Group environments generated by peer-led teaching versus peer-based talking may have varying consequences on programme effectiveness and outcomes. Though outside the scope of this research, this distinction between education and dialogue and the subsequent impacts on programme effectiveness for long-term behaviour change are worth exploring in the future.

Although TESFA was primarily designed to mitigate the effects of child marriage on adolescent girls, this study found considerable evidence that TESFA contributed to decreased community acceptance and adoption of early and child marriage. Five years post-TESFA, girls and community members had maintained supportive attitudes and actions towards delayed marriage. In considering the implications of these findings on programmes aimed at reducing child marriage, the authors developed a hypothesis relating to social capital theory [20]. Girls Groups provided married girls with recognition, acceptance and visibility in their homes and communities that they are unable to achieve individually. This social capital earned through group participation and economic activity accumulates over time into a "ban", that girls then draw from to engage in what could be deemed socially risky behaviors, such as delaying marriage. TESFA’s effectiveness at changing and sustaining improvements to community perceptions and practices of child marriage spotlights the need for programmes to embed gender-transformative processes in community-oriented platforms. Reviews of the characteristics of interventions effective in reducing harmful SRH practices determined that sustained outcomes require creating platforms, such as ongoing dialogues, that encourage a community to critically examine and shift its traditions [21, 22]. Although TESFA is an adolescent-focused programme, its consideration of the community’s role and use of community members' power in its design was crucial to its success. Because of SAA members’ active engagement and actions in the programme, behavioural changes enacted by married girls were perceived positively despite not reflecting traditional expectations. The meaningful participation and active role that TESFA afforded community members through SAA groups, as primary links to and supporters of Girls’ Groups, created a sense of responsibility, ownership, and recognition by girls’ families, wider community, and local government. Per the findings that spousal support was essential to girls’ autonomy, peer-based ASRH programmes need to further engage husbands to better ensure sustainability of outcomes post-implementation. In their assessments of peer education programmes, Michielsen et al. and Chandra-Mouli et al. suggest integrating peer education into holistic interventions for increased effectiveness [16, 18]. Having a cadre of committed adults with the awareness and skills to promote social norms allowing for girls’ choice, voice, and mobility, was a critical factor in sustaining programme impact and facilitating the effectiveness of Girls Groups. As Cislaghi et al suggest is a necessary condition for scale, this diffusion of programming to influential persons within girls’ communities paved the way for organic scale-up of the TESFA model [23]. This study’s findings on the sustained impacts of TESFA on child marriage through an inclusive dialogue approach lend further support to the need for effective platforms for holistic community engagement.

One strategy for creating or integrating holistic interventions with community platforms is to leverage existing community structures [24]. TESFA’s savings and loan component was familiar to the community because of Ethiopia’s traditions of *equb* [8]. Ethiopia has a long history of informal savings institutions where members make contributions to a joint fund that they can borrow against for personal or business ventures [8]. Although women commonly participate in *equb*, they tend to be past adolescence. An exploration of the characteristics of *equb* members in rural and urban areas of Ethiopia showed that heads of household typically participate and that the average age of female participants is 24 years [25]. TESFA extended the opportunity to partake in a savings structure to married adolescent girls, a population typically excluded from *equb*. By then delivering SRH curriculum within these girls-only savings groups, and simultaneously providing programming to others in the community via SAA groups, TESFA created a platform that leveraged existing Ethiopian structures to deliver an integrated and holistic intervention. Kirstos’ study of *equb* participation on Ethiopian women’s lives suggests that structures would be more effective if coupled with support services [26]. This study validates that with its findings that while savings structures fueled a continued purpose for peer groups to come together, the solidarity within and supportiveness of group rules towards members influenced their motivations to sustain.

### Considerations for sustainable and scalable peer-based programme design

The factors that impacted the sustainability and scalability of TESFA’s peer-based solidarity groups in the 5 years since the programme ended are summarized in Additional file 2: Figure S2.

After analyzing these factors, the authors recommend the following considerations for implementers designing peer-based programmes to ensure sustainability and scalability:

- Environment of solidarity: Intragroup dynamics are central to the success, scale-up, and sustainability of TESFA’s peer-based design. Creating safe spaces to enable girls’ solidarity and share their ideas encourages adoption of new behaviours and motivates them to sustain changes.
- Holistic community intervention: Community support enables girls to engage in positive SRH behaviours and sustain and scale their groups. Support from husbands is particularly essential for married girls’ mobility, participation, and agency.
- Culturally relevant curriculum: Availability of ample curriculum/dialogue topics is vital for sustainability and scale. When developing curriculum, including people with lived experience and contextual knowledge increases programme relevance. TESFA was heavily informed by Ethiopian female health workers, some of whom had experienced early marriage and were familiar with the local context and needs.
- Shared facilitation responsibility: Group facilitators’ commitment and leadership are key to sustainment and scale-up. Where facilitators were unable to continue in their roles, dependence on a few trained facilitators compromised group sustainability and active engagement of other members. Future peer-based approaches might consider utilizing a rotating facilitator model [27].
- Transitional economic opportunity: Although TESFA’s VSLA was pivotal for group sustainability, it lacked a longer-term accountability mechanism. Facilitating groups’ transition from small-scale savings and loans processes to accessing *equb* or other financial services is important to consider.
- Inclusive group procedures: Flexible processes that can be adjusted to suit members’ needs including meeting schedules, norms, and bylaws, contribute to group ownership and longevity.

### Limitations

Because the TESFA programme was not originally designed with a sustainability intention, the recommendations shared in this paper are based on an evaluation of the conditions that contributed to an organic scale-up and sustainment of the programme. This study’s exploration of the determinants of sustainability and scalability of peer-based ASRH programmes is limited in that it assessed outcomes for a particular programme over a finite period. Although TESFA was successful in achieving some level of sustainability and scale, it is unknown whether that will last beyond 5 years post-implementation without additional outside investment. As a result, study findings may be limited in their applicability for sustaining programmes for longer than 5 years. In addition, this paper attempts to present participants’ objective reflections about the TESFA programme. However, congruent with norms in Ethiopian culture, dissenting opinions were generally uncommon within focus groups. Study teams attempted to correct for this by establishing group agreements before discussions to create open and unbiased group environments. A final limitation is that this study did not collect data on or consider the impacts of girls aging out of participation in TESFA during the post-implementation period.

## Conclusion

With a growing global population of adolescents, designing effective interventions for their unique needs will be central to ensuring their sexual and reproductive health rights. Married adolescent girls are particularly susceptible to sexual and reproductive risk because of early marriage and demand special attention in adolescent programming. TESFA’s methodology is effective in improving the lives of married adolescent girls and mitigating some of the harmful effects of child marriage. Outcomes related to girls’ solidarity and mobility, contraceptive use, and institutional delivery were sustained over time. The observed organic replication and scale-up of the TESFA model by adolescents beyond its intended beneficiaries reflects the model’s ability to address the distinct needs of adolescents and its potential to scale widely across Ethiopia. With its demonstrated ability to improve the lives of married girls, TESFA provides a tested model for successful, scalable, and sustainable adolescent reproductive health programme delivery through peer-based solidarity groups.

## Supplementary Information

**Additional file 1. **FGD and IDI Guides. Two sample focus group discussion guides and one in-depth interview guide used with Girls Groups and community members.

**Additional file 2: Figs. S1, S2. ** Illustrating a timeline of TESFA activities and summarizing the facilitators and barriers for scale and sustainability.

## Data Availability

The datasets used and analyzed during the current study are available from the corresponding author on reasonable request.

## Abbreviations

ACIPH: Addis Continental Institute of Public Health
ASRH: Adolescent sexual and reproductive health
EE: Economic empowerment
FGDs: Focus group discussions
GG: Girls Group
HIV: Human immunodeficiency virus
IDIs: In-depth interviews
RAs: Research assistants
SAA: Social analysis and action
SRH: Sexual and reproductive health
TESFA: Towards Economic and Sexual/reproductive Health Outcomes for Adolescent Girls
VSLA: Village Savings and Loan Associations
