# Supplementary material for: Building sustainable and scalable peer-based programming: promising approaches from TESFA in Ethiopia
Source: Reprod Health. 2022 Jun 13;19(Suppl 1):55. doi: 10.1186/s12978-021-01304-7 (PMC9195188; doi:10.1186/s12978-021-01304-7)
Supplement: Supplementary file 1 — Additional file 1. FGD and IDI Guides. Two sample focus group discussion guides and one in-depth interview guide used with Girls Groups and community members. [file 12978_2021_1304_MOESM1_ESM.pdf]

BMC Reproductive Health – Special Supplement on Ethiopia

Article Title – Successful, Scalable and Sustainable: Using the TESFA programme as a model for peer-based programming

Additional File 1

Focus Group Discussion Guide: Dissolved original Girls Group members

1. Think back to your time on the Girls Group during the TESFA program. What did you see as the purpose of your group?
   Probe: Did you feel like the group had accomplished this purpose at the end of the program?
   Probe: During the TESFA program, was your understanding that the group would stop once the program ended?
   a. How has your participation in a Girls Group affected your life?
      Probe: Is there anything that is still different in your life now because of the program, or is everything the same as it was before the program?
      Probe: What do you consider to be the most valuable aspects of your participation in the group?
   b. What changes, if any, have you noticed in your community since the TESFA program ended?
      Probe: How do you feel about no longer participating in a group?

2. I would like to know more about the process that your group went through before dissolving. When did your group stop meeting?
   a. What was the impetus for dissolving your group?
      Probe: Did your group dissolve as soon as the program ended, or did it continue for some time after that and then eventually stop?
   b. How did the decision to dissolve first come about?
      Probe: Was the decision to stop meeting made by the whole group together, or did one person lead that process?
      Probe: What role did your group's original facilitators play in this process?
   c. Was there any debate/discussion between group members about whether to maintain or dissolve the group?
      - [If yes] What were some of the factors that were discussed?

      Probe: Once the final decision to dissolve your group had been made, did everyone in your group agree with that or were there some people who wanted to maintain the group?
      Probe: How did you feel about your group coming to an end at that time?
      Probe: Did anyone from your original group join another group once yours dissolved?

3. I want to understand why some groups that were originally part of the TESFA program have since dissolved. What were the reasons why your group stopped meeting?
   a. What made it difficult to maintain the group?
   b. Describe your group's relationship with the SAA group.
      Probe: What kind of support did you receive from them?
      Probe: How did the SAA group affect your group's decision to dissolve?
   c. Describe your group's relationship with ORDA.
      Probe: What kind of support did you receive from them?
      Probe: How did ORDA affect your group's decision to dissolve?

d. Was there any external pressure to stop meeting as a group?
   Probe: What role did people outside your group (eg. Community members, husbands, mothers in law, village leaders etc.) play in the decision to dissolve your group?
   Probe: How did your mother-in-law feel about your participation in the group?
   Probe: How did your husband feel about your participation in the group?

4. How was your group's decision to dissolve influenced by environmental factors?
   a. How did participating in the group affect your ability to fulfill other responsibilities?
      Probe: How much time did group participation involve in any given month?
   b. How difficult was it for you to travel to and from group meetings?
      Probe: Did you ever feel unsafe during your travel?
   c. How did the drought affect your ability to participate in the group?
      Probe: How was your family affected by the drought?
      Probe: How did your priorities change following the drought?

5. Given what you have shared so far about why your group stopped, I would like to talk about some things that could have helped maintain your group. What conditions would have been necessary for your group to have continued?
   a. What was different between your group and other groups in the area that continued?
      Probe: Do you think there is value to maintaining a group?
   b. Who all would your group have needed support from to continue?
      Probe: What else would you have needed for your group to continue?

6. If your group was to get back together, how would you feel about that?
   a. What characteristics of your original group would you maintain?
      Probe: Would you use the same curriculum?
      Probe: Would you maintain the Sexual and Reproductive Health component?
      Probe: Would you maintain the Economic Empowerment component?
   b. What would you change?

7. Are there any other thoughts about your experiences in your group that you'd like to share?

<u>Focus Group Discussion Guide: Auto-replicated Girls Group members</u>

1. One thing we hope to understand is how groups that were not originally part of the TESFA program were formed. Can you tell me how the group that you are a part of came to exist?
   a. Who had the idea of getting together in a group?
      Probe: Did your group start with someone/people who were members of an existing TESFA group before?
   b. Why is the idea of meeting in a group similar to TESFA interesting to you?
      Probe: What did you think the benefits would be?
      - Have you experienced these same benefits?
   c. How supportive is your family to the idea of meeting in these groups?
   d. The original TESFA groups were organized in a way that each group had two facilitators who led the trainings for the group. Is this also how your group was organized?
   e. The original TESFA groups all focused on married girls who were younger than 19. What is the age of the girls in your group?

2. Now I would like to ask you about how the groups function. Can you tell me what kind of things your group does?
   a. How often does your group usually meet?
   b. What topics are usually discussed in the group meetings?
      Probe: How are topics for discussion determined?
   c. Does your group usually have some sort of training included as part of most meetings?
      [If yes] Does the training follow a specific curriculum?
         [If yes] Where did the trainer/facilitator get the curriculum from?
      [If yes] What topics are usually included in that training? (Ask them to list these out as specifically as they can).

3. In your group, is there usually a discussion or training about sexual and reproductive health matters, such as use of antenatal services or family planning?
   [If yes, proceed to Question 5]
   [If no, provide some specific examples of topics that were included in the original TESFA SRH curriculum and check if they report discussing any of the topics]

4. How useful did you generally find this training to be?
   Probe: How has it changed the way that you feel about using family planning?
   Probe: How has it changed how much you use reproductive health services?
   Probe: How has it changed your control of when and how many children you have?
   Probe: How has it changed your communication with your husband about family planning?

5. In your group, are members required to contribute to a group fund and borrow money?
   [If yes] What do you usually use the money you borrow for?

6. In your group, is there usually a discussion or training about how to manage savings, starting a small business, etc.?
   [If yes] How useful did you generally find this training to be?
   Probe: How has it changed the way that you manage your finances, such as how much money you save and what you are saving for?
   [If no, provide some specific examples of topics that were included in the original TESFA EE curriculum and check if they report discussing any of the topics]

7. What was it about the TESFA group that made you want to have your own group?
   Probe: What were the things that made it easy for you to start this group?
   Probe: What were the things that made it hard to start this group?
   - What can be done to make it easier for girls to create their own TESFA-like groups?
   - What kind of challenges has your group or groups like yours faced?
     Probe: How did the group overcome the challenge?

8. Since you began your group, what, if any, changes have you observed in your community?
   a. What changes have you noticed in communication within households?
      Probe: Would you share a decision in the household with your husband? What are decisions that are still not shared?
   b. What changes have you noticed in your ability to go places outside of your home?
   c. Do husbands punish or insult their wives in your community?
      Probe: What are the reasons for punishing or insulting?

   d. What changes have you noticed in the practice of early marriage?
      Probe: How did this change come about?
   e. What changes have you noticed in the practice of female genital mutilation/cutting?

9. If TESFA is started in other communities, which parts of TESFA do you think would be most beneficial for those girls?

10. Do you have any suggestions and recommendations on the overall TESFA project that we have not discussed so far?

<u>In-depth Interview Guide: Health extension worker</u>

1. I would like to start by asking you about what you know about the former TESFA project and the change in the community because of the project. Can you tell me what have you heard of the TESFA project?

2. What do you think are the sustained benefits that Girls Groups got from the TESFA project?
   a. Do you think the benefits found at the end of the program are still existing today in those same communities?
   b. What impacts do you think have been sustained since the project ended?

3. Now I would like to ask you about the effect that you feel the TESFA project had on the lives of the girls who participated in the program. Here I am discussing those girls who participated in the original TESFA project. First, let us focus on sexual and reproductive health. What changes have you seen as a service provider?
   a. Do the girls know of and/or come for antenatal care visits?
      - Do TESFA girls come seeking these services more or less than others?
   b. Do the girls still know and /or practice different type family planning?
      - Do they come seeking the services more or less than others?
   c. Do the girls know and practice (plan to) having institutional delivery?
      - Do they come seeking these services more or less than others?
   d. Do the girls know how to prevent HIV/STIs?
      Probe: Do they know about the testing?
      - Do they come seeking these services?

4. Now I would like to talk about the effect that you feel the TESFA project had on the norms and expectations that affect girls' lives. Here I am discussing those girls who participated in the original TESFA project. What changes have you seen in the community?
   a. What has changed within the households of TESFA girls?
      Probe: What household decisions do girls share in with their husbands?
      - Do girls typically have to get/receive permission to use family planning?

      Probe: What are decisions that are still not shared?
   b. What changes have you noticed in girls' ability to go places outside of home?
      Probe: Do girls typically have to get/receive permission to come to the clinic to see you?
   c. What changes have you noticed in the practice of early marriage?
      Probe: How do you think this change came about?
   d. What changes have you noticed in the practice of female genital cutting?
      Probe: How do you think the community feels about this practice?

5. Now I would like to talk about the effect that you feel the TESFA project had on girls' economic empowerment. Here I am discussing those girls who participated in the original TESFA project. How do you think savings have affected the lives of the TESFA girls?

6. Aside from the changes in health and economic activities, what do you feel was the most important change that happened because of the TESFA project?
   a. What was the most important change for the participants?
   b. What was the most important change for the community?
   c. Were there bad things that came from the TESFA project?
      [If needed] *Probe*: Were there people who were unhappy with the project?

7. Do you see any changes in sexual and reproductive health practices among girls who were not in the original TESFA program?
   - [If any] Do you think TESFA influenced these changes?

8. What do you think are the most important things in the TESFA reproductive health curriculum?
   a. If we did the program again, is there anything you would add to the health content?
      Probe: Is there anything that you have seen in other programs or have thought would be helpful for young married girls that we could include?

9. Do you have any suggestions and recommendations on the overall TESFA project that we have not discussed so far?
