# Supplementary material for: Building sustainable and scalable peer-based programming: promising approaches from TESFA in Ethiopia
Source: Reprod Health. 2022 Jun 13;19(Suppl 1):55. doi: 10.1186/s12978-021-01304-7 (PMC9195188; doi:10.1186/s12978-021-01304-7)

BMC Reproductive Health – Special Supplement on Ethiopia

Article Title – Successful, Scalable and Sustainable: Using the TESFA programme as a model for peer-based programming

Additional File 2

Figure 1: Timeline of CARE's implementation and subsequent evaluations of the TESFA programme between 2010 and 2010

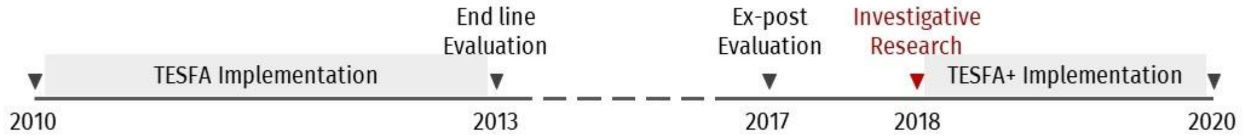

Figure 2: Facilitators and barriers of scale and sustainability of TESFA adolescent sexual and reproductive health peer-based solidarity groups between 2013 and 2018

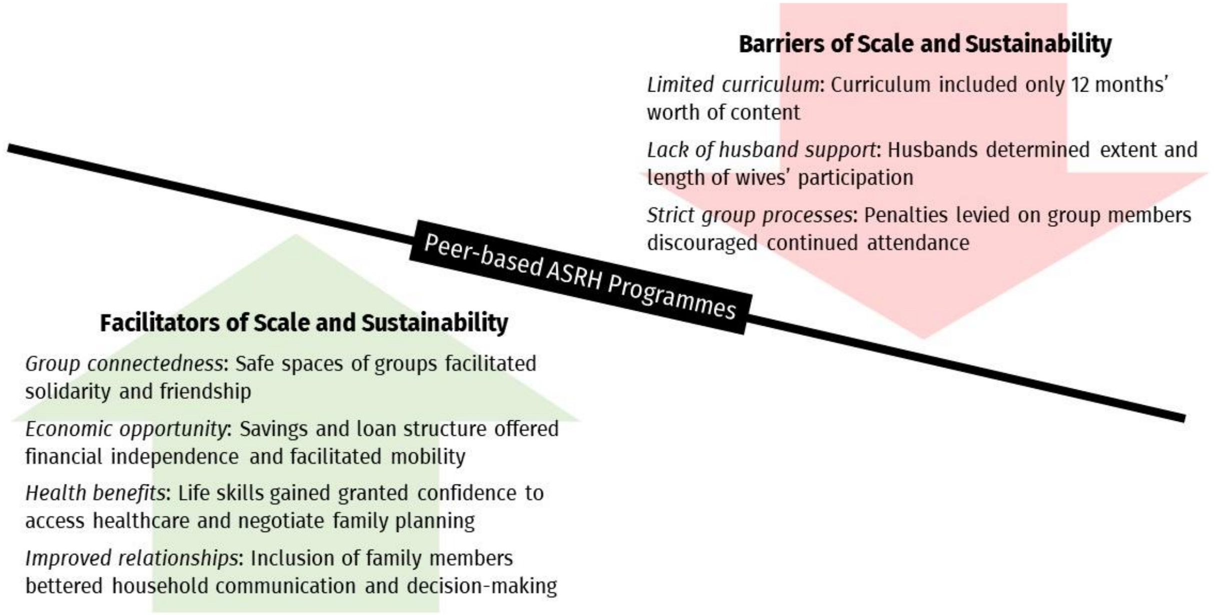

Supplement: Supplementary file 2 — Additional file 2: Figs. S1, S2. Illustrating a timeline of TESFA activities and summarizing the facilitators and barriers for scale and sustainability. [file 12978_2021_1304_MOESM2_ESM.pdf]
